# Nicotinic alpha 7 receptor agonists EVP-6124 and BMS-933043, attenuate scopolamine-induced deficits in visuo-spatial paired associates learning

**DOI:** 10.1371/journal.pone.0187609

**Published:** 2017-12-19

**Authors:** Michael R. Weed, Joseph Polino, Laura Signor, Mark Bookbinder, Deborah Keavy, Yulia Benitex, Daniel G. Morgan, Dalton King, John E. Macor, Robert Zaczek, Richard Olson, Linda J. Bristow

**Affiliations:** 1 Genetically Defined Diseases and Genomics, Bristol-Myers Squibb Company, Wallingford, CT, United States of America; 2 Pharmaceutical Candidate Optimization, Bristol-Myers Squibb Company, Wallingford, CT, United States of America; 3 Discovery Chemistry, Bristol-Myers Squibb Company, Wallingford, CT, United States of America; Scripps Research Institute, UNITED STATES

## Abstract

Agonists at the nicotinic acetylcholine alpha 7 receptor (nAChR α7) subtype have the potential to treat cognitive deficits in patients with Alzheimer’s disease (AD) or schizophrenia. Visuo-spatial paired associates learning (vsPAL) is a task that has been shown to reliably predict conversion from mild cognitive impairment to AD in humans and can also be performed by nonhuman primates. Reversal of scopolamine-induced impairment of vsPAL performance may represent a translational approach for the development of nAChR α7 agonists. The present study investigated the effect of treatment with the acetylcholinesterase inhibitor, donepezil, or three nAChR α7 agonists, BMS-933043, EVP-6124 and RG3487, on vsPAL performance in scopolamine-treated cynomolgus monkeys. Scopolamine administration impaired vsPAL performance accuracy in a dose- and difficulty- dependent manner. The impairment of eventual accuracy, a measure of visuo-spatial learning during the task, was significantly ameliorated by treatment with donepezil (0.3 mg/kg, i.m.), EVP-6124 (0.01 mg/kg, i.m.) or BMS-933043 (0.03, 0.1 and 0.3 mg/kg, i.m.). Both nAChR α7 agonists showed inverted-U shaped dose-effect relationships with EVP-6124 effective at a single dose only whereas BMS-933043 was effective across at least a 10 fold dose/exposure range. RG3487 was not efficacious in this paradigm at the dose range examined (0.03–1 mg/kg, i.m.). These results are the first demonstration that the nAChR α7 agonists, EVP-6124 and BMS-933043, can ameliorate scopolamine-induced cognitive deficits in nonhuman primates performing the vsPAL task.

## Introduction

Compounds which activate nicotinic acetylcholine receptors of the α7 receptor subtype (nAChR α7) have received considerable attention because of their potential to treat cognitive symptoms in patients with schizophrenia or Alzheimer’s disease (AD) [1, 2]. In preclinical species, many nAChR α7 agonists have been shown to reliably improve cognition in a variety of different models assessing different cognitive domains. Thus in normal subjects, nAChR α7 agonists have been shown to improve attention [3, 4] and the retention of episodic memory in rodents [5–8] and to improve working memory in nonhuman primates [9–12]. In addition these agents have been shown to reverse N-methyl-D-aspartate (NMDA) receptor antagonist-induced deficits in attentional set shifting [13–15] and object recognition memory in rodents [6, 16], and to alleviate working memory deficits in nonhuman primates [17]. In humans NMDA receptor antagonists such as ketamine and phencyclidine (PCP) produce symptoms that resemble those seen in schizophrenia patients including psychotomimetic effects, negative symptoms and cognitive impairment [18, 19] NMDA antagonists are therefore commonly used as a pharmacological approach to model schizophrenia-like symptoms in animals.

With respect to AD, many studies have examined the ability of nAChR α7 agonist treatment to reverse cognitive impairment induced by administration of the nonselective muscarinic AChR antagonist scopolamine. This agent has been used extensively in rodents to model the cholinergic deficits associated with aging and AD [20, 21]. Treatment with nAChR α7 agonists has been shown to reverse scopolamine-induced deficits in object recognition memory retention [7, 22], spatial working memory [23, 24]; and avoidance learning and memory in rodents [7, 25]. Additional studies in aged animals have also shown improvements in spatial learning and working memory in the Morris water maze task [15, 25]. Finally, nAChR α7 agonist treatment of aged 3X Tg AD mice, at a time when subjects showed marked AD related pathology (i.e. β amyloid plaques, neuro-inflammation and neurofibrillary tangles), significantly improved spatial learning and memory in the Morris water maze task, object recognition memory and contextual fear memory demonstrating efficacy in a disease relevant model for this mechanism [26].

The development of novel agents for the treatment of cognitive impairment is greatly facilitated by the use of translational tests that can be applied across species to examine different cognitive domains. In this regard, the CAmbridge Neuropsychological Testing Automated Battery (CANTAB) provides a translational platform that can be used to examine attention, executive function and visual learning and memory in both humans and nonhuman primates [27–30]. In humans, CANTAB has been extensively validated in different patient populations including AD and schizophrenia patients who exhibit poor performance on multiple tests when compared to age matched, healthy subjects [31–34]. Of note, impaired performance of the visuo-spatial Paired Associates Learning (vsPAL) test, which requires subjects to learn an association between an abstract shape and its spatial location on a computer monitor, is observed in patients with mild cognitive impairment (MCI) and worsens in AD; indeed impaired vsPAL performance may predict progression from MCI to AD [32–35]. Functional imaging studies also show abnormal hippocampal activation patterns in MCI patients performing the vsPAL task consistent with the hypothesis that hippocampal dysfunction drives the cognitive deficits seen in the early stages of AD [36]. Furthermore recent studies show that treatment-naive AD patients, receiving a single 5 mg dose of the acetylcholinesterase inhibitor donepezil, have improved performance of the CANTAB vsPAL task [37]. Thus CANTAB vsPAL task performance can reliably detect cognitive impairment in MCI and AD patients and is sensitive to the administration of cholinergic therapeutic agents.

In nonhuman primates performance of the CANTAB vsPAL task is impaired by acute scopolamine treatment, a pharmacological approach used to model AD-like cholinergic deficits [38, 39]. In humans, scopolamine treatment also impairs vsPAL performance in most [40, 41] but not all studies reported [42]. In addition, recent studies in humans demonstrate that scopolamine-impaired performance of the CogState vsPAL task can be ameliorated by donepezil treatment [40]. These results suggest that this approach may have translational utility for the development of novel AD therapeutics. Given the potential of nAChR α7 agonists in this regard, the present study was conducted to examine these agents in the nonhuman primate scopolamine vsPAL model. Specifically, we examined the effect of acute treatment with BMS-933043 [13, 43], EVP-6124 [22] or RG3487 [15], in comparison to donepezil, in scopolamine-treated cynomolgus monkeys trained to perform the CANTAB vsPAL task. At the time these studies were initiated, EVP-6124 and RG3487 were the most advanced clinical nAChR α7 agonists and were therefore ideal comparators for characterization of BMS-933043 in a translational model.

## Materials and methods

### Subjects

Thirteen experimentally naïve male cynomolgus monkeys (Macaca Fasicularis; 3–5 years of age; 5.0–7.5 kg at the beginning of the study) served as subjects. Subjects were housed in pairs, separated during testing (Monday through Friday) and re-paired after feeding in the afternoon. Subjects were fed standard monkey chow (Harlan Teklad Global 20% protein Primate Diet 2050) in sufficient quantities to ensure normal growth while maintaining sufficient motivation to perform the cognitive testing [30]. Water was continuously available except during behavioral testing. Fresh fruit, toys and foraging devices were routinely provided in the monkey colony rooms. Laboratory animal care was according to Public Health Service Policy on the Humane Care and Use of Laboratory Animals and the Guide for the Care and use of Laboratory Animals, [44]. The protocol and experiments were approved by the Wallingford Animal Care and Use Committee of the Bristol-Myers Squibb Company.

### Apparatus

For behavioral testing subjects were seated comfortably in restraint chairs (Primate Products, Immokalee, FL), and placed in individual, sound attenuated chambers (Med-Associates, St. Albans, VT), equipped with lights, a white noise generator and a monitoring camera. A touch-sensitive computer monitor was within easy reach and session events were controlled by a Monkey CANTAB test station; (Lafayette Instruments, Lafayette, IN) and Whisker Control [45]. Following correct responses, a dispenser delivered 190 mg banana-flavored pellets (Bioserv, Frenchtown PA).

### Behavioral procedure

Ten subjects were trained to perform the CANTAB vsPAL test as previously described [38, 46]. Briefly, the test required the animal to associate a stimulus (abstract shape) with a given spatial location on the computer screen (one of the four corners; S1 Fig). Ten trials each of increasing difficulty (2-, 3- or 4- stimuli) were presented. Trials began with the ‘sample phase’ where stimuli are presented and touched sequentially in their respective locations with a 1 sec delay between stimuli. Following 5 sec where the screen was blank, the choice phase began. The same stimulus was presented in all four corners of the screen for all difficulty levels. Touching the stimulus in the sample location, within 30 sec, resulted in delivery of a food pellet. After a 1 sec screen blank another stimulus was presented in all four corners. If the animal touched the correct location for all shapes in the choice phase on the first attempt the trial was recorded as ‘correct on the initial attempt’. Touching a stimulus in an incorrect location (or failing to touch in 30 sec) resulted in a 10 sec screen blank (time-out) and the same trial was repeated from the beginning. The same trial could be repeated up to 5 additional times.

If the animal learned the sequence by the 6th attempt, the trial was recorded as ‘eventual correct’, or ‘incorrect’ if not. Failure to respond within 30 s of each stimulus presentation was considered an ‘omission.’ Omissions counted in the number of attempts per trial, but did not count as ‘errors’ and did not affect the percent correct responding for a given difficulty level. If an animal failed to respond in a given condition, data for that condition was omitted for that animal and interpolated with the group mean for statistical analysis. Additional measures were the percent completion (the proportion of trials on which both sample and choice responses were made), and response latency (time from sample onset to the screen touch in the choice phase, averaged across difficulty conditions).

### Drugs

**Fig 1:**
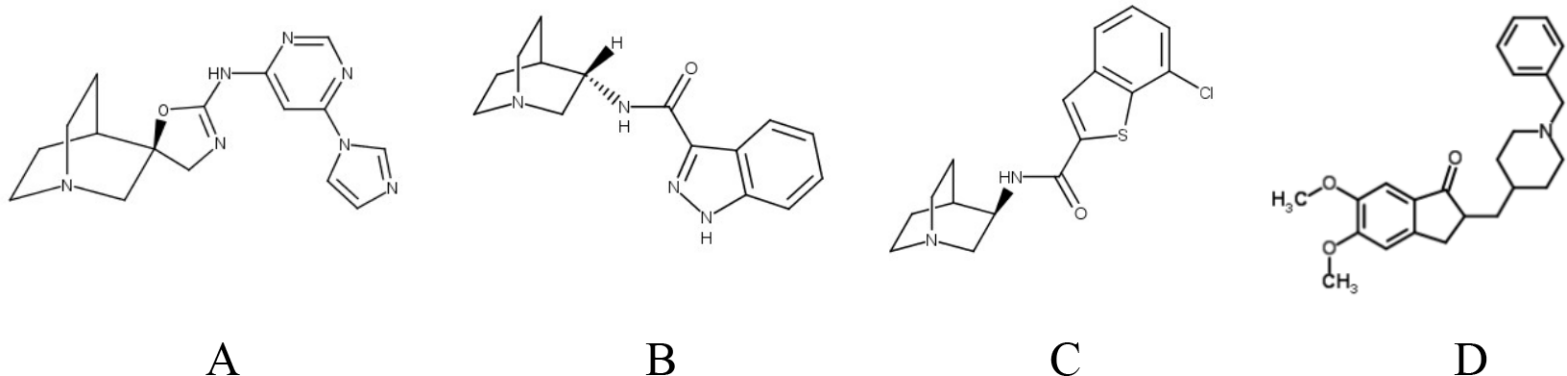
Chemical structures of A) BMS-933043, B) RG3847, C) EVP-6124 and D) donepezil.

Donepezil hydrochloride (HCl) was purchased from Sigma-Aldrich. Scopolamine HCl, EVP-6124 HCl (Encenicline; (R)-7-chloro-N-(quinuclidin-3-yl)benzo[b]thiophene-2-carboxamide HCl)[22], RG3487 HCl (also known as R3487, MT-666 and MEM 3454; N-[(3S)-1-Azabicyclo[2.2.2]oct-3-yl]-1H-indazole-3-carboxamide HCl)[47] and BMS-933043 ((2*R*)-*N*-(6-(1*H*-imidazol-1-yl)-4-pyrimidinyl)-4'*H*-spiro[4-azabicyclo[2.2.2]octane-2,5'-[1,3]oxazol]-2'-amine) [13, 43] were synthesized at Bristol Myers Squibb. The chemical structures of these compounds are shown in Fig 1. Doses cited refer to the free base form of the compounds. Vehicles were sterile normal saline for scopolamine, donepezil, R3487 and EVP-6124, with sterile normal saline at pH 4.0 for BMS-933043. All drugs were administered intramuscularly (i.m.) in a dosing volume of 0.1–0.2 ml/kg. The dose range examined for each compound was as follows: Donepezil (0.03–0.3 mg/kg), BMS-933043 (0.03–1.0 mg/kg), EVP-6124 (0.003–1.0 mg/kg) and R3487 (0.03–1.0 mg/kg).

### Drug testing

Drug testing was initiated once animals had achieved asymptotic and stable baseline performance on the 4 stimuli condition using the eventual correct measure. Drug testing was conducted on Mondays and Thursdays. An additional baseline session was conducted on Tuesdays. The first six subjects to reach stable performance were included in the scopolamine dose-response study. Additional animals were included in later studies as their performance improved.

In the scopolamine dose-response study all subjects received a single i.m. injection of scopolamine or saline 15 min prior to the session. For the initial scopolamine-challenge study with donepezil, a dose of 0.0056 mg/kg scopolamine was chosen for all animals, and animals were habituated to receive two injections of saline (0.1 ml/kg i.m.) at 15 and 30 min prior to the session. Some animals demonstrated larger impairments than others to the 0.0056 mg/kg dose; therefore, in subsequent studies the dose of scopolamine was individually titrated for each subject to achieve at least a 10% reduction in eventual accuracy in the 4- stimulus condition on two sessions with a range of 15% between those sessions. Scopolamine dose was re-titrated prior to each study and scopolamine was administered no more than three times in any two-week period during testing. There was a washout period of at least 2 weeks between studies. This schedule of scopolamine administration was well tolerated. Over the two-year period of this evaluation the mean dose of scopolamine increased from 0.0056 mg/kg (range = 0.0045–0.0067 mg/kg) to 0.009 mg/kg (range = 0.0045–0.012 mg/kg). The chronological order of drug testing and mean scopolamine dose for each study was as follows: donepezil (0.0056 mg/kg scopolamine), BMS-933043 (0.0051 mg/kg scopolamine), RG3487 (0.0087 mg/kg scopolamine), EVP-6124 study 1 (0.009 mg/kg scopolamine) and EVP-6124 study 2 (0.009 mg/kg scopolamine).

Performance after vehicle+vehicle or vehicle+scopolamine treatment was determined before, during and after the dose response function for each compound and their respective means used in the data analysis (mean of at least 3 determinations). On days when compound testing occurred, approximately half the cohort received one dose and the remainder another dose of the same compound and therefore dose order was staggered between animals. With the exception of EVP-6124, all other compounds were tested in a single dose response study. EVP-6124 was tested in two independent studies because the first study with 0.03, 0.1, 0.3 and 1.0 mg/kg failed to cover the lower end of clinically relevant exposures. Therefore, a second EVP-6124 study with 0.003 and 0.01 mg/kg was conducted. Scopolamine dose titration, vehicle+vehicle and vehicle+scopolamine were determined separately for each study.

### Statistical analyses

**Fig 2:**
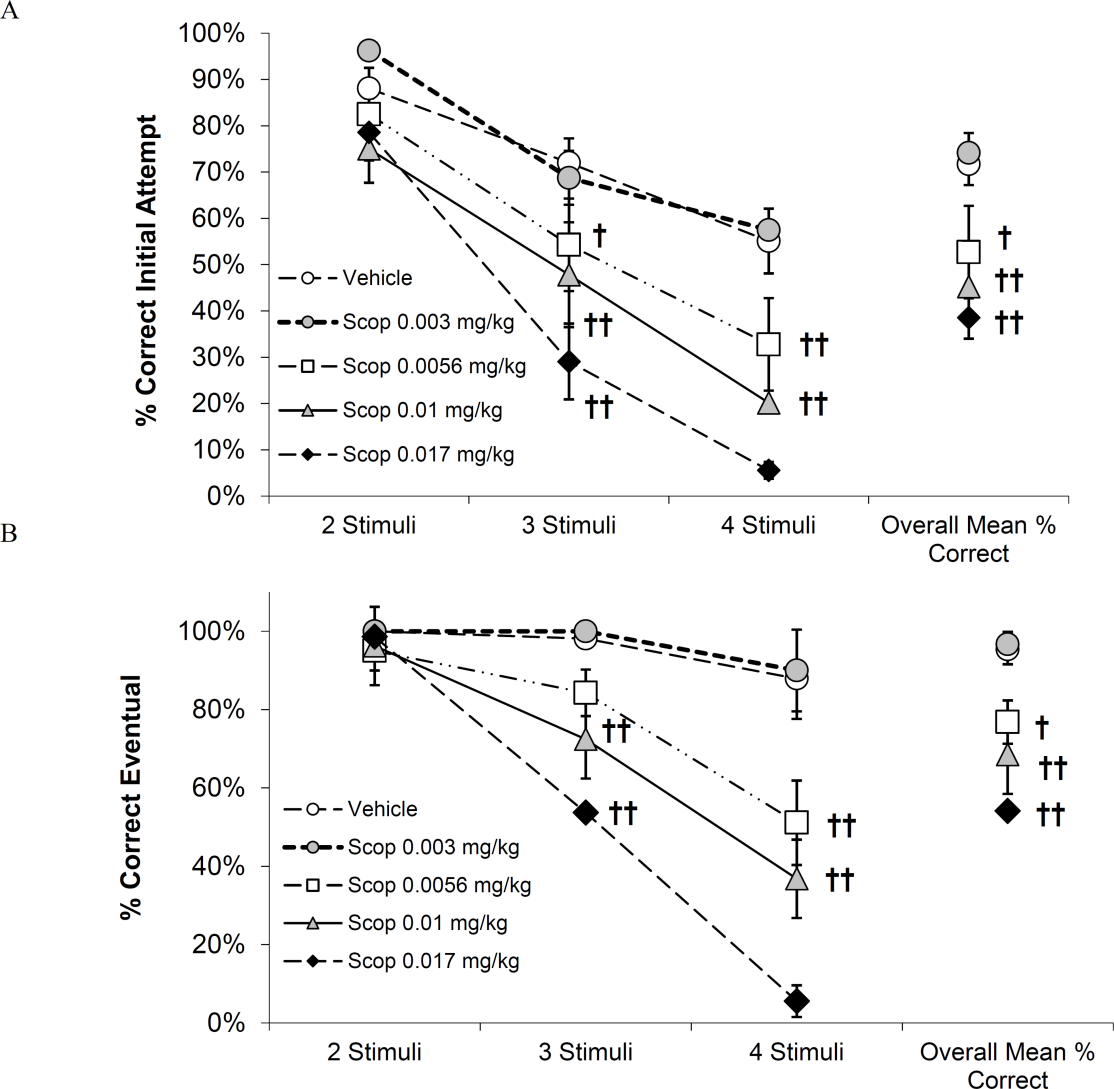
**Effect of scopolamine treatment on A) % correct responses at the initial attempt and B) % eventual correct responses achieved within 6 attempts.** Results are presented as the mean ± SEM (N = 8) % initial or eventual correct responses for trials with 2-, 3- or 4- stimuli and were analyzed by 2 way RM-ANOVA followed by Holm-Sidak’s post hoc analysis; † *p<0*.*05* and †† p*<0*.*01* compared to vehicle treatment. Results at the 4- stimuli condition after 0.017 mg/kg scopolamine were not analyzed because only 3/8 subjects responded.

**Fig 3:**
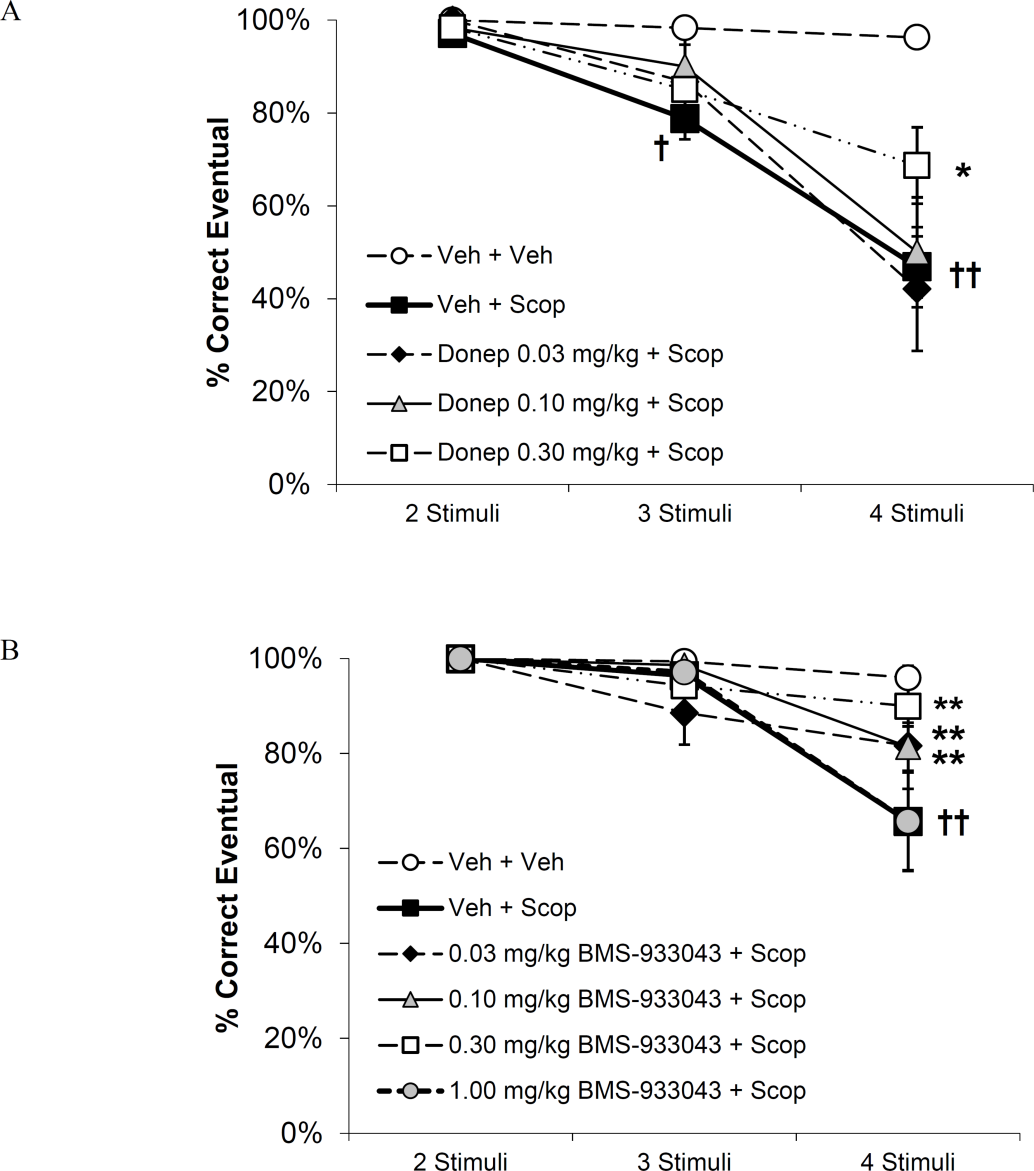
**Effect of treatment with A) donepezil (N = 6) or B) BMS-933043 (N = 7) on scopolamine-induced impairment of learning in the vsPAL task.** Results are presented as the mean ± SEM % eventual correct responses achieved within 6 attempts after presentation of trials with 2-, 3- or 4- stimuli. Results were analyzed by 2 way RM ANOVA followed by Holm-Sidak’s post-hoc analysis; † *p<0*.*05* and †† *p<0*.*01* compared to vehicle/vehicle treatment; * *p<0*.*05* and ** *p<0*.*01* compared to vehicle+scopolamine treatment.

**Fig 4:**
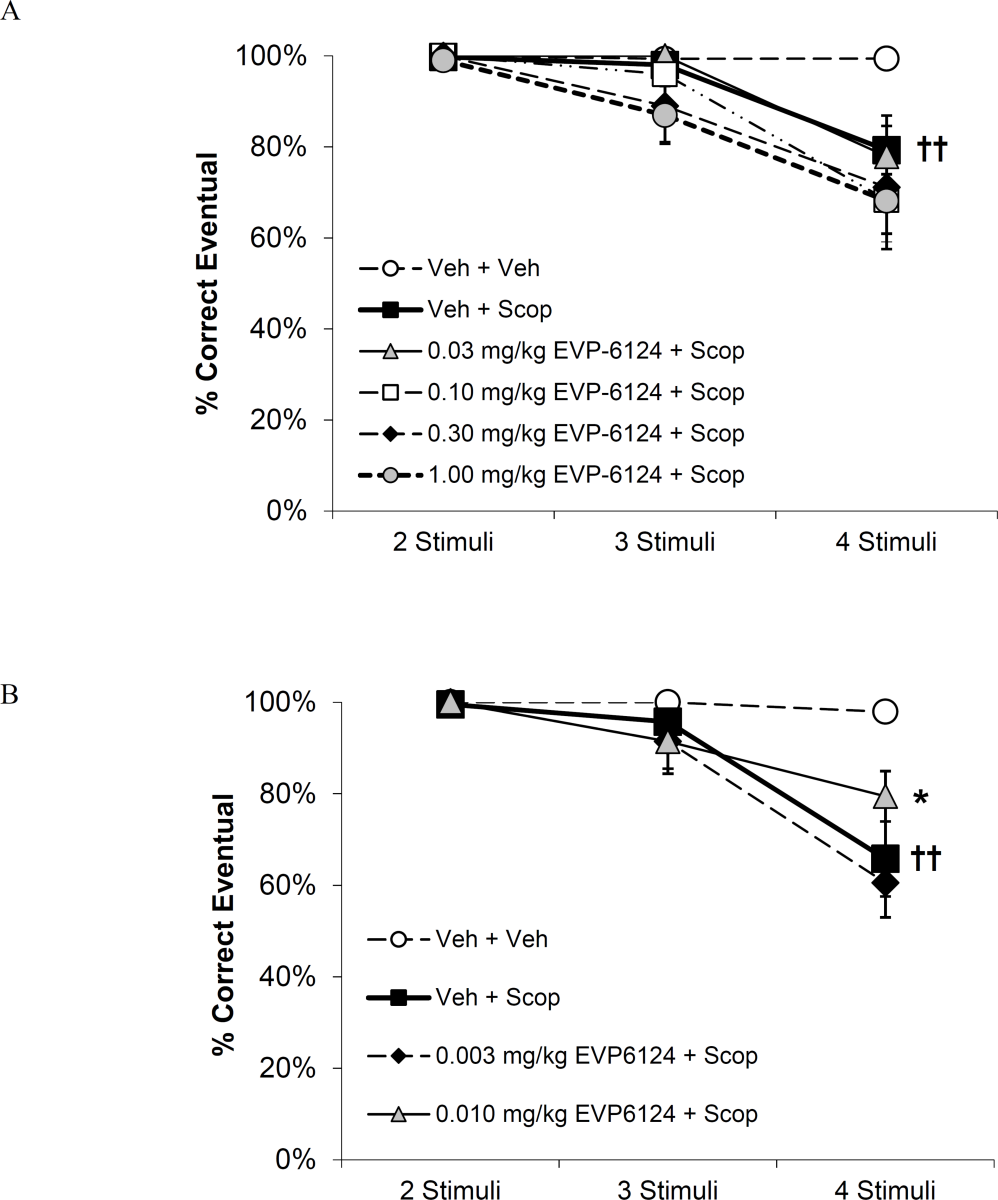
**Effect of treatment with EVP-6124 at A) 0.03–1.0 mg/kg (N = 10) or B) 0.003–0.01 mg/kg (N = 7) on scopolamine-induced impairment of learning in the vsPAL task.** Results are presented as the mean ± SEM % eventual correct responses achieved within 6 attempts after presentation of trials with 2-, 3- or 4- stimuli. Results were analyzed by 2 way RM ANOVA followed by Holm-Sidak’s post-hoc analysis; †† *p<0*.*01* compared to vehicle/vehicle treatment; * *p<0*.*05* compared to vehicle+scopolamine treatment.

**Fig 5:**
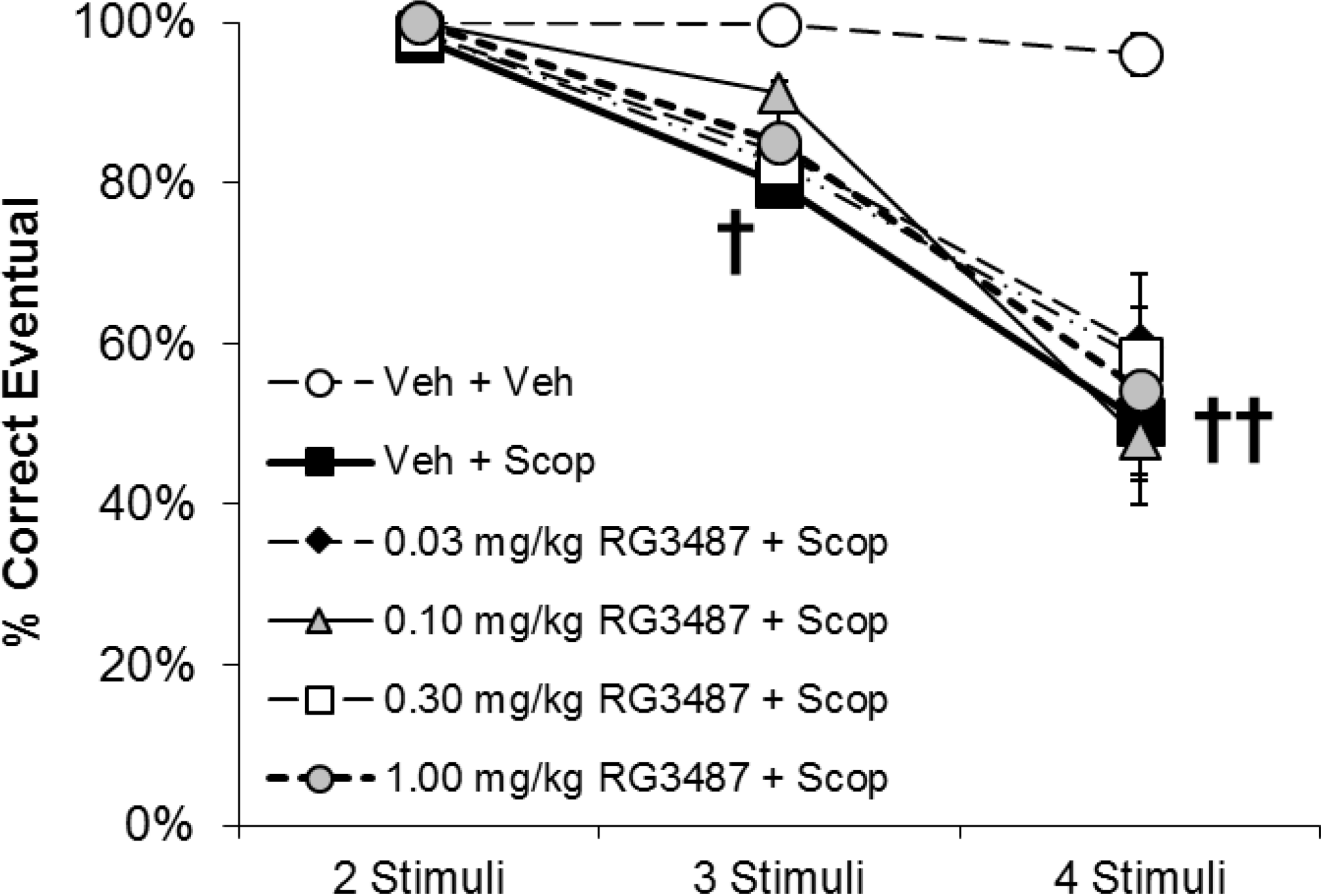
Effect of treatment with RG3487 on scopolamine-induced impairment of learning in the vsPAL task (N = 8). Results are presented as the mean ± SEM % eventual correct responses achieved within 6 attempts after presentation of trials with 2-, 3- or 4- stimuli. Results were analyzed by 2 way RM ANOVA followed by Holm-Sidak’s post-hoc analysis; † *p<0*.*05* and †† *p<0*.*01* compared to vehicle/vehicle treatment.

Accuracy measures (i.e. percent initially correct and percent eventually correct) were analyzed with repeated measures analysis of variance (RM-ANOVA) with the two within-subject factors of difficulty (mean of number of stimuli per trial and overall mean) and treatment. Latencies to respond in all choice phases and percent task completed for the entire session were analyzed by one way RM ANOVA. Post-hoc analysis was conducted using Holm-Sidak’s method to compare all treatment conditions with vehicle+scopolamine responses (drug studies) or scopolamine doses with vehicle treatment (scopolamine dose response study). Statistical tests were performed using OriginPro v 9.1 (OriginLab Co., Northampton, MA). Missing data were filled with the mean of the respective cell to enable RM ANOVA computation. Means following each difficulty level and the overall session mean are presented for scopolamine (Fig 2). For simplicity of presentation, overall session means are not presented in figures for test compounds (Figs 3–5) but are included in the supplemental data (S1 File) along with all applicable data for this manuscript. Performance following scopolamine 0.017 mg/kg treatment at the 4- stimuli condition resulted in excessive missing data (i.e. only 3/8 monkeys responded) and post-hoc tests were not conducted on that condition.

Effect size was calculated for the treatments which statistically differed from scopolamine using an online tool from Becker, 2000 (http://www.uccs.edu/~lbecker/). Cohen’s d values of >0.2, >0.5 and >0.8 are described using the conventions of small, medium, and large effects, respectively [48].

### Pharmacokinetic studies

For all compounds, plasma pharmacokinetic studies were conducted in a separate cohort of cynomolgus monkeys (N = 2–3 subjects per dose) before beginning vsPAL testing to help guide dose selection. Blood samples were obtained with the animals awake and sitting in a primate chair. For most test sessions with BMS-933043 and EVP-6124, plasma samples were also taken from a subset of vsPAL subjects (N = 4–5) immediately after completion of testing (mean (SD) sample collection time = 69.8 (9.8) min post injection).

### Plasma concentration analysis

Plasma concentrations of test compounds were determined using LC-MS/MS assays. In brief, 50 μL plasma and sample containing compounds was extracted by protein precipitation method with 150 μL acetonitrile containing an internal standard. A 5 μL sample of supernatant was injected into the LC-MS/MS system. Analytes were separated by reversed phase chromatography and detection of each analyte was achieved through selected reaction monitoring mode of mass spectrometry. Samples were quantified by calculated peak area ratio of analyte and internal standard against a calibration curve.

## Results

### Scopolamine effects on vsPAL performance

Cynomologus monkeys trained to perform the vsPAL task showed the expected difficulty-dependent reduction in initial attempt accuracy under vehicle treatment conditions (Fig 2A). Repeated attempts of these trials improved eventual accuracy to asymptotic levels (Fig 2B). Scopolamine treatment produced a dose-dependent impairment in vsPAL performance as shown by a reduction in both initial accuracy (Fig 2A) and eventual accuracy (Fig 2B). RM ANOVA confirmed the impairment of initial accuracy with significant main effects of task difficulty (F_3,21_ = 198.8; *p*< 0.001), treatment (F_4,28_ = 16.4; *p*< 0.001) and their interaction (F_12,84_ = 2.8; *p =* 0.003). Post-hoc analyses showed significant reductions in initial accuracy for the 3- stimuli conditions following treatment with scopolamine at doses of 0.0056, 0.01 and 0.017 mg/kg (*p*< 0.05, *p*< 0.01 and *p*< 0.01 respectively; Fig 2A). Similarly, 0.0056 and 0.01 mg/kg scopolamine impaired performance of the 4- stimuli condition at the *p*< 0.01 level. In contrast, performance accuracy at the least difficult, 2- stimuli condition was not significantly impaired at any dose of scopolamine tested (Fig 2A). RM ANOVA also confirmed the impairment of eventual accuracy with significant main effects of task difficulty (F_3,21_ = 139.6; *p*< 0.001), treatment (F_4,28_ = 31.3; *p*< 0.001) and their interaction (F_12,84_ = 11.1; *p*< 0.001). Further post-hoc analysis showed significant reductions in eventual accuracy for both the 3- and 4- stimuli conditions (*p*< 0.01 for 0.0056 and 0.01 mg/kg; Fig 2B). Eventual accuracy at the 2- stimuli condition was not significantly impaired by any dose of scopolamine tested (Fig 2B).

Latency to respond in the choice phase was not significantly affected by scopolamine treatment (F_4,28_ = 0.72; *p*> 0.05; S1 Table). In contrast, a significant reduction in the percent of task completed was observed in subjects treated with scopolamine at doses above 0.003 mg/kg (F_4,28_ = 11.0; *p*< 0.001; S1 Table). This pattern was consistent throughout this series of studies, and scopolamine impaired percent task completion consistently, while affecting latency to respond only once (vehicle+scopolamine differed from vehicle/vehicle in the second EVP-6124 study). Interestingly, treatment with donepezil or any of the three nAChR α7 agonists did not alter the scopolamine-induced reduction in percent task completed (S1 and S2 Tables). S2 Table presents RM ANOVA details for response latency and percent of task completion for all compounds tested.

### Treatment effects on initial accuracy

Scopolamine impaired performance on the initial correct measure in each study. However, none of the treatments significantly reversed the scopolamine impairment on initial attempt accuracy (S3 and S4 Tables). In contrast, the highest doses of EVP-6124 exacerbated the scopolamine-induced impairment in initial accuracy. RM ANOVA of initial accuracy confirmed significant main effects of task difficulty (F_3,27_ = 199.4; *p*< 0.001), treatment (F_5,45_ = 14.0; *p*< 0.001) and their interaction (F_15,135_ = 5.9; *p*< 0.001). Post-hoc analysis confirmed that vehicle+scopolamine treatment impaired initial accuracy compared to vehicle/vehicle treatment at both the 3- and 4- stimuli conditions (S4 Table). Additionally, impairment of initial accuracy in the 3- stimuli condition following EVP-6124+scopolamine was significantly greater than that of vehicle+scopolamine following 0.3 and 1.0 mg/kg EVP-6124. Trends towards further impairment in the 4- stimulus condition did not reach significance (S3 and S4 Tables).

### Treatment effects on eventual accuracy

Donepezil significantly reduced the impairment of eventual accuracy produced by scopolamine at the most difficult, 4- stimuli task condition (Fig 3). Two-way RM ANOVA confirmed significant main effects of task difficulty (F_3,15_ = 44.9; *p*< 0.001), treatment (F_4,20_ = 4.9; *p* = 0.006) and their interaction (F_12,60_ = 6.4; *p*< 0.001; Fig 3A). Post-hoc analysis confirmed that treatment with vehicle+scopolamine impaired eventual accuracy compared to vehicle/vehicle treatment in the 4- stimuli condition (Fig 3A). The impairment seen after scopolamine treatment on the 4- stimuli condition was significantly reversed by donepezil at the 0.3 mg/kg dose (Fig 3A) with a Cohen’s d of 1.2 consistent with a large effect size. Mean plasma concentrations of donepezil, determined in satellite animals, increased in relation to dose. The average plasma concentration at the 0.3 mg/kg dose was 90.3 nM determined 60 min post-administration (i.e. a time point coinciding with the completion of behavioral testing) (S5 Table).

BMS-933043 significantly reduced the impairment of eventual accuracy produced by scopolamine at the most difficult, 4- stimuli task condition. RM ANOVA of eventual accuracy confirmed significant main effects of difficulty (F_3,18_ = 15.1; *p*< 0.001), treatment (F_5,30_ = 5.3; *p* = 0.001) and their interaction (F_15,90_ = 3.2; *p*< 0.001; Fig 3B). Post-hoc analysis confirmed that treatment with vehicle+scopolamine impaired eventual accuracy compared to vehicle/vehicle treatment at the 4- stimuli task condition (Fig 3B). This impairment was significantly reversed in subjects treated with BMS-933043 at doses of 0.03, 0.1 and 0.3 mg/kg but not when treated with the highest dose of 1 mg/kg indicating a U-shaped dose-effect relationship (Fig 3B). The Cohen’s d values were 0.64, 0.73 and 1.2, indicating medium to large effect sizes following administration of 0.03, 0.1 and 0.3 mg/kg BMS-933043, respectively. Mean plasma concentrations of BMS-933043, determined in experimental subjects on completion of behavioral testing, increased in relation to dose with improved eventual accuracy observed over the range of 13.3 to 139.5 nM (S5 Table).

EVP-6124 treatment was examined in 2 separate studies in order to evaluate an extended dose range (Fig 4). Results from the first study showed that EVP-6124, at doses ranging from 0.03–1.0 mg/kg, failed to improve eventual accuracy and worsened initial accuracy in scopolamine treated subjects (S3 Table). RM ANOVA of eventual accuracy confirmed significant main effects of task difficulty (F_3,27_ = 21.6; *p*< 0.001), treatment (F_5,45_ = 3.8; *p* = 0.006) and their interaction (F_12,60_ = 6.4; *p*< 0.001; Fig 4A). Post hoc analysis confirmed that treatment with vehicle+scopolamine impaired eventual accuracy relative to vehicle/vehicle treatment at the 4- stimuli condition and that EVP-6124 treatment failed to alter this impairment (Fig 4A). The second study examined lower doses of EVP-6124 and showed a significant improvement in eventual accuracy at the 0.01 mg/kg dose. RM ANOVA of eventual accuracy confirmed significant main effects of task difficulty (F_3,18_ = 16.5; *p*< 0.001), treatment (F_3,18_ = 15.6; *p*< 0.001) and their interaction (F_9,54_ = 6.3; *p*< 0.001; Fig 4B). Post-hoc analysis confirmed that treatment with vehicle+scopolamine impaired eventual accuracy compared to vehicle/vehicle treatment at the 4- stimuli condition (Fig 4B) and that this was significantly attenuated after treatment with 0.01 mg/kg EVP-6124 (Fig 4B). The Cohen’s d after 0.01 mg/kg EVP-6124 was 0.82, consistent with a large effect.

In contrast to the effects seen at higher doses, worsening of initial accuracy was not observed in study 2 (S3 Table). Overall the results from both studies show that EVP-6124 exhibits a steep inverted-U shaped dose-effect relationship with improvements in eventual accuracy detected at a single dose only. Mean plasma concentrations of EVP-6124 determined after the behavioral session, increased in relation to dose with improved eventual accuracy observed at 1.6 nM (S5 Table). Worsening of initial accuracy was associated with plasma concentrations ranging from 53.1 to 170.2 nM (S5 Table).

Treatment with RG3487, at doses ranging from 0.03–1.0 mg/kg, failed to improve eventual accuracy in scopolamine treated subjects (Fig 5). RM ANOVA of eventual accuracy confirmed significant main effects of task difficulty (F_3,21_ = 26.0; *p*< 0.001), treatment (F_5,35_ = 5.5; *p*< 0.001) and their interaction (F_15,105_ = 3.3; *p*< 0.001; Fig 5). Post-hoc analysis confirmed that treatment with vehicle+scopolamine impaired eventual accuracy compared to vehicle/vehicle treatment at the 4- stimuli condition (Fig 5) and that RG3487 treatment did not alter this effect (Fig 5). Mean plasma concentrations of RG3487, determined in satellite animals 60 min post-administration (i.e. a time point corresponding to completion of behavioral testing) ranged from 58.7 to 450.0 nM at the 0.1–1.0 mg/kg dose range (S5 Table).

**Fig 6:**
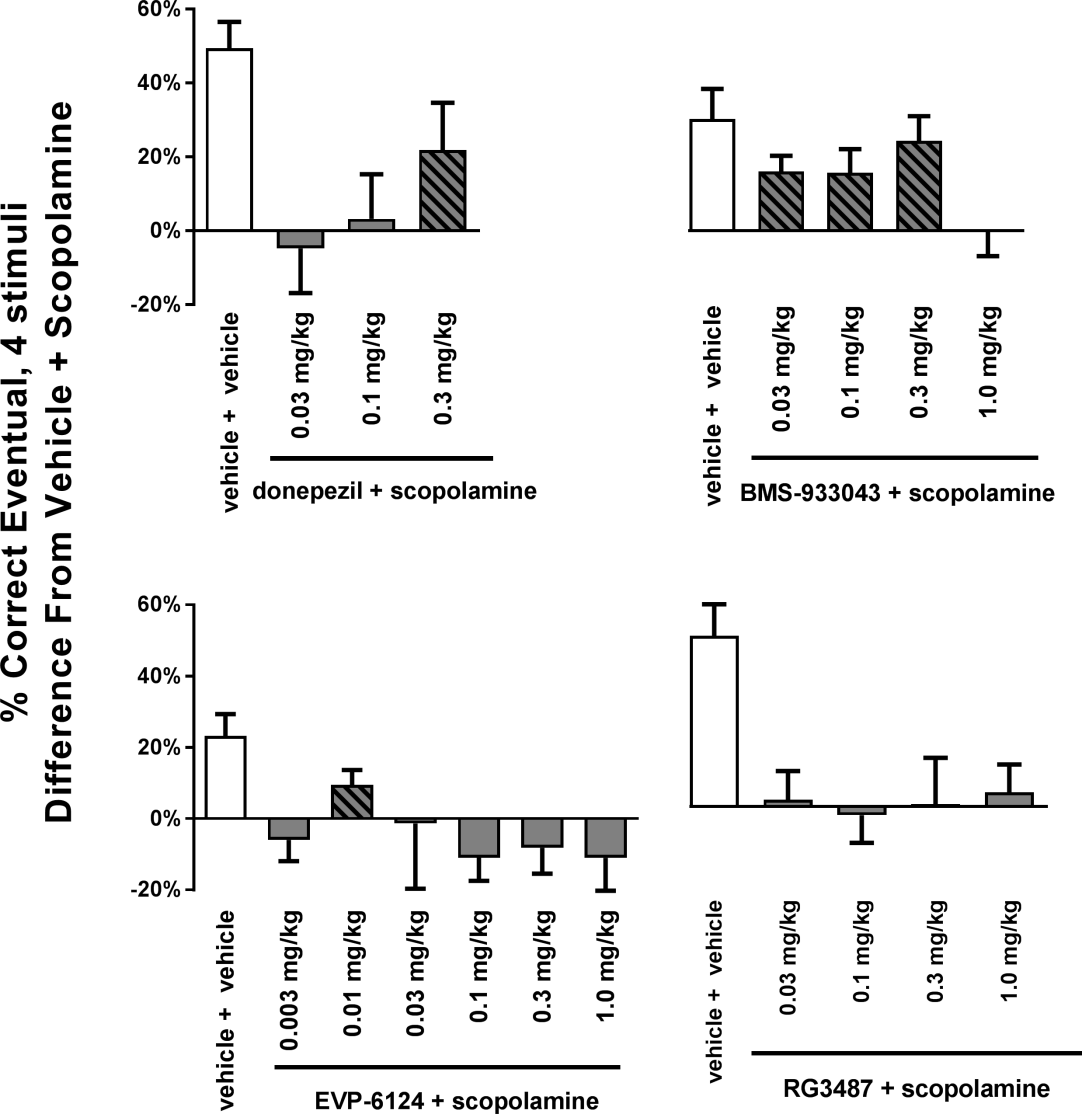
Comparison of the dose-effect curves to reverse scopolamine-induced impairment of learning in the vsPAL task (% correct eventual). For each subject, treatment effects on the % correct eventual response were expressed as a difference from the vehicle+scopolamine condition, and group means and SEMs were replotted for side-by-side comparisons. Y axis is percent change from vehicle+scopolamine condition. X axis indicates test condition. Hatched bars indicate a test combination differed significantly from vehicle+scopolamine in post-hoc tests from RM ANOVA dose-response functions (e.g. at *p*<0.05 or *p*<0.01).

The effects of donepezil and nAChR α7 agonist treatments on the percent correct eventual response within the 4- stimuli condition are presented together in Fig 6 to illustrate the shape of the dose-effect functions. For each study, the results are presented as the difference in the % correct eventual between the vehicle+scopolamine condition and other treatments (i.e., vehicle+vehicle or compound+scopolamine). With EVP-6124, the vehicle+vehicle and the vehicle+scopolamine results were averaged from the two studies to enable presentation of both results on same graph. Fig 6 illustrates the dose ranges across which donepezil, EVP-6124 and BMS-933043 attenuated scopolamine’s effects (hatched bars, based on their respective RM ANOVAs reported above). Fig 6 also illustrates the study to study variability in the scopolamine impairment, (e.g. the difference between vehicle+vehicle and vehicle+scopolamine for each study) which ranged from 49.4% in the donepezil study and 20.5% in the first EVP-6124 study.

### Treatment effects on task completion and latency to respond

Consistent with results from the scopolamine dose-response function, small but statistically significant reductions in percent task completion were observed after individually-titrated scopolamine doses. None of the treatments significantly altered the effect of scopolamine on this measure (S1 and S2 Tables). Latency to respond in the choice phase was typically unaffected by vehicle+scopolamine treatment or drug+scopolamine treatment (S1 and S2 Tables). While a significant increase in vehicle+scopolamine response latency was seen in the second study with EVP-6124 this was not different from latencies seen in EVP-6124+scopolamine conditions consistent with a lack of effect of nAChR α7 agonist treatment on latency to respond (S1 and S2 Tables).

## Discussion

The present study shows that treatment with the acetylcholinesterase inhibitor, donepezil or the nAChR α7 agonists, BMS-933043 or EVP-6124, improved performance of the CANTAB vsPAL task in scopolamine-treated cynomolgus monkeys. Consistent with previous studies, treatment with scopolamine produced a difficulty-dependent impairment of accuracy on both the initial attempt and the eventual correct response, measures that reflect the formation of a stimulus-location association and incremental strengthening of the association, i.e. learning, respectively [38, 39]. Treatment with donepezil, BMS-933043 or EVP-6124 selectively improved the eventual correct response indicating a specific effect on the learning component of the vsPAL task. Furthermore, this effect was observed in the absence of changes in measures of psychomotor speed or motivation to work suggesting a true pro-cognitive effect. To our knowledge this is the first report demonstrating that treatment with nAChR α7 agonists can improve learning in a scopolamine-induced deficit model in nonhuman primates.

While BMS-933043 and EVP-6124 significantly improved vsPAL learning in scopolamine-treated subjects, neither agent affected the accuracy of the first attempt (initial accuracy). This was somewhat unexpected, as both compounds have been shown to improve recognition memory in rodents [13–15]. However, the first attempt of vsPAL is not a recognition task *per se* as it requires recall for an association between a stimulus and a location, rather than recognition of a previously seen stimulus. Instead the nAChR α7 agonists reversed scopolamine’s effect on ‘eventual correct’, improving performance on the learning component of the vsPAL model.

Both BMS-933043 and EVP-6124 showed inverted-U shaped dose-effect curves with improvement at lower doses and a loss of efficacy observed at higher doses. This was particularly striking for EVP-6124 where efficacy was observed at a single dose only, while BMS-933043 retained efficacy across at least a 10 fold dose/exposure range. The potential for steep inverted-U shaped dose-response curves is well recognized for this mechanism and is thought to reflect receptor desensitization. However, the effects of receptor desensitization on the shape of the dose-response curve are not well understood as studies have reported that multiple nAChR α7 agonists, including EVP-6124, retain efficacy after repeated dosing in clinical and preclinical models [15, 26, 49–51]. Unfortunately, chronic dosing was beyond the scope of the present study.

Inverted-U shaped dose-response functions present significant challenges for clinical development when testing of a broad dose range in patients may be unrealistic [52]. The inverted-U shape was also seen with nAChR α7 agonists in NMDA receptor antagonist-induced deficit assays used to model the cognitive and sensory processing impairment seen in schizophrenia patients. Thus while BMS-933043 improved both S(+)-ketamine-induced N40 gating deficits and MK-801-induced set shifting deficits in rats, efficacy was lost at higher doses in both assays [13]. The efficacious plasma BMS-933043 concentrations ranged from 50–1,860 nM and 146–1,360 nM in the two rat models respectively [13]. In the nonhuman primate scopolamine vsPAL model, efficacy was observed at plasma BMS-933043 exposures ranging from 13–139.5 nM, but lost at plasma concentrations of 493 nM, indicating that the exposure-effect curve is similar to but slightly left-shifted compared with rodent models. In the case of EVP-6124, previous studies in rats have also shown inverted-U shaped dose-effect curves in the novel object recognition (NOR) model, both with respect to improvement in 24 hour recognition memory and reversal of scopolamine-induced impairment [22, 53]. In the rodent NOR studies efficacy was retained over a 10 fold dose range whereas in the nonhuman primate scopolamine vsPAL model EVP-6124 was effective at a single dose only. However, at the maximally effective dose in rats, plasma EVP-6124 concentrations (1–2 nM) were similar to those achieved at the efficacious dose in the present study (1.6 nM). Thus, in scopolamine deficit models, the inverted-U shaped dose-effect relationship for EVP-6124 is much steeper in nonhuman primates compared to rodents but appears to be centered around similar optimal plasma concentrations.

EVP-6124 was chosen for these studies as it has also progressed to clinical testing enabling comparison of preclinical results to effects seen in humans. In healthy volunteers, single oral doses ranging from 1–180 mg showed linear pharmacokinetics with C_max_ values ranging from 0.6–100 ng/ml (1.8–312 nM) achieved 5–8 hours after dosing [54]. Subjects were also tested for performance of the Digital Symbol Substitution Test (DSST), a neuropsychological test that examines attention, psychomotor speed and speed of processing [55]. Acute administration of EVP-6124 improved DSST performance and also showed an inverted-U shaped dose-response with maximal effects observed at the 20 mg dose corresponding to a plasma Cmax of 10.4 ng/ml (32 nM) [54]. In contrast, results from a Phase II study in schizophrenia patients suggest that improvements in cognition, as reflected by composite performance scores across several tests in the CogState battery or MATRICS Consensus Cognitive Battery (MCCB), can be achieved at low doses (0.27 and 0.9 mg) producing steady state trough plasma concentrations of 1.6–5.5 nM [56]. Thus the effective dose range of EVP-6124 appears to differ in healthy humans and schizophrenia patients. Interestingly, efficacious exposures from preclinical studies, including the present study (1.6 nM) align most closely with schizophrenia patient exposures for EVP-6124 (1.6–5.5 nM) [56].

In contrast to BMS-933043 and EVP-6124, no reversal of scopolamine-induced impairment of vsPAL performance was observed in subjects treated with the nAChR α7 agonist RG3487. These results were unexpected given preclinical studies showing that RG3487 improved object recognition memory retention in naive rats, spatial learning in aged rats and attentional set shifting in chronic PCP-treated rats [15]. While a variety of dosing routes were used across these studies, maximal plasma exposures in separate pharmacokinetic studies ranged from 24–591 ng/ml (89–2,186 nM) at oral doses (1–10 mg/kg) reported to be efficacious in the rat NOR model [15]. RG3487 was also effective in nonhuman primate cognition models however these results have been reported in conference abstracts only and have not been fully published. Nevertheless in unimpaired subjects, improved performance of the object retrieval (response inhibition) and delayed match to sample (DMTS; working memory) tasks was reported with inverted-U shaped dose-effect curves seen in both cases [57]. The efficacious plasma RG3487 concentration range, at the minimally effective dose for pro-cognitive effects across species/models, was cited as 50–200 nM [58]. In addition intramuscular injection of 0.3 mg/kg RG3487 was reported to improve working memory in aged Rhesus monkeys performing the DMTS task [59]. In the present study we tested RG3487 across the 0.03–1 mg/kg im dose range and achieved exposures (predicted and measured) ranging from ~ 15–450 nM, which overlap with doses/exposures reported in other preclinical studies. In addition, despite variability in the scopolamine-induced impairment in vsPAL performance, the magnitude of scopolamine impairment was similar in the RG3487 and donepezil studies, where a significant reversal by donepezil was observed. Nevertheless, given the slightly more modest scopolamine deficit seen in studies testing EVP-6124 and BMS-933043, further investigation may be warranted to confirm the lack of activity of RG3487 in this model. Understanding compound levels in the brain, and receptor occupancy may also enlighten this issue, but these measures were beyond the scope of the present study.

A limitation of these studies was that the scope of the project did not allow for repeated dosing of each agonist dose (only a single determination), and did not allow for testing of the compounds on their own (e.g. without scopolamine); therefore, the effect of nAChR α7 agonists on vsPAL performance *per se* is unclear. However, notwithstanding the limitations discussed above, the different nAChR α7 agonist treatment effects observed in the scopolamine vsPAL model do not appear to be easily explained by differences in *in vitro* pharmacology. Thus all compounds show potent binding affinity to rat and/or human nAChR α7 with Ki values in the 1–10 nM range depending on the specific radioligand used [13, 15, 22]. With respect to intrinsic efficacy, all agents show a partial agonist profile *in vitro* although the degree of agonism may differ depending on the measure, the experimental system and the potential for receptor desensitization on successive applications of the test agent. Direct comparison of BMS-933043 and EVP-6124, using voltage clamp electrophysiology on HEK293 cells expressing human nAChR α7, showed that EVP-6124 has higher intrinsic activity using both the peak current response (32% versus 24%) and net charge (136% versus 78%) to define the response relative to acetylcholine [13]. EVP-6124 may also have higher intrinsic activity compared to RG3487; in xenopus oocytes expressing human nAChR α7 the maximal peak current elicited by EVP-6124 was 80% of the acetylcholine response as compared to 63% reported for RG3487 [15, 22]. Thus the steep inverted-U shaped dose-effect curve seen for EVP-6124 may in part reflect the higher intrinsic efficacy of this agent. It is also interesting that we observed a worsening of initial accuracy at higher doses of EVP-6124 that was not seen at any dose of BMS-933043 or RG3487. RG3487 has also been tested in QM7 cells expressing human nAChR α7; the maximal peak current and net charge was 69% and 53% respectively relative to acetylcholine [15]. The degree of partial agonism is therefore higher or lower than BMS-933043 depending on the measure suggesting this is unlikely to explain the difference between these 2 agents. Finally, it should be noted that RG3487 and EVP-6124 are also potent 5-HT3 receptor antagonists [15, 22] and that treatment with 5-HT3 antagonists is reported to improve recognition memory in macaques [60]. Recent studies with tropisetron, a non-selective nAChR α7 agonist and 5-HT3 receptor antagonist, also show improved working memory in aged macaques performing the DMTS task [11]. While this additional pharmacological activity may complicate interpretation of the effects seen *in vivo*, studies in rodents have confirmed that the pro-cognitive effects of RG3487 and EVP-6124 are blocked by the selective nAChR α7 antagonist methyllycaconitine [15, 22]. In a similar manner, *in vitro* and *in vivo* results support the role of nAChR α7 in mediating the pro-cognitive effects of tropisetron [11, 61]. Importantly, BMS-933043 is highly selective for nAChR α7 versus the 5-HT3 receptor confirming that nAChR α7 agonism is sufficient to produce pro-cognitive effects [13]. Thus additional pharmacology at the 5-HT3 receptor seems unlikely to account for the different profiles observed for these agents in the scopolamine vsPAL model.

An important consideration for the present study was the selection of the vsPAL procedure as a model suitable for translation from nonhuman primates to humans. In this regard, recent studies in healthy humans have shown that scopolamine produces a robust reduction in vsPAL performance accuracy that was significantly improved by co-treatment with the acetylcholinesterase inhibitor donepezil [40]. An important note is that similar to the results of the current study in monkeys, donepezil only partially reversed scopolamine’s impairment in the learning component of vsPAL in humans [40]. The results seen in the present study are therefore consistent with those reported in humans performing this task. Other cholinergic agents reported to be efficacious in the scopolamine vsPAL model in nonhuman primates include the M1 receptor selective positive allosteric modulator PQCA (1-((4-cyano-4-(pyridine-2-yl)piperidin-1-yl)methyl-4-oxo-4 H-quinolizine-3-carboxylic acid) [39] and the nAChR α7 agonists tested in the present study, EVP-6124 and BMS-933043. Unfortunately human data for either M1 modulators or nAChR α7 agonists in the scopolamine vsPAL task are not currently available and the utility of the model as a translational approach remains to be determined. This is particularly relevant for the nAChR α7 mechanism which, thus far, has proven disappointing in the clinic despite the extensive body of preclinical literature supporting the approach. This disconnect was recently explored by meta-analyses to compare the effect size for cognitive improvement in rodents with results from patients [52]. The analyses included several different nAChR α7 agonists and showed that collectively, these agents produced large effect sizes in rodent cognition models such as the NOR and Morris water maze test. Large treatment effect sizes (i.e. Cohen’s D values >8) were also seen at maximally efficacious doses of EVP-6124 and BMS-933043 in the present study indicating that the magnitude of the pro-cognitive effect is consistent across rodents and nonhuman primates. In contrast, evaluation of results from patients showed that nAChR α7 agonist treatment effect sizes were small and of uncertain clinical significance [52]. It should be noted, however, that the majority of the results included in the analyses were from schizophrenia patients; only 3 out of 18 studies were conducted in AD patients with 2 reporting only composite cognition scores. Results for EVP-6124 in AD patients could not be included because of insufficient data disclosure; this agent completed Phase II studies but was discontinued in Phase III due to serious gastrointestinal side effects [49, 50]. Interestingly, the incorporation of human paradigms, with increased face validity to animal paradigms, was proposed as a strategy to improve the clinical translation for nAChR α7 agonists. In this regard, the scopolamine vsPAL model may serve as a suitable bridging model to the support the further development of novel agents like BMS-933043.

In conclusion, treatment with donepezil significantly improved learning of stimulus/location pairs in scopolamine-treated nonhuman primates performing the vsPAL task, effects similar to those reported in healthy humans. Treatment with the nAChR α7 agonist, BMS-933043, was also effective and exhibited a broader dose-effect relationship compared to EVP-6124. The results suggest that the scopolamine vsPAL model may have utility as a translational approach to support the transition of novel therapeutics into early clinical development.

## Supporting information

S1 Fig Schematic of vsPAL procedure. Example of the most difficult trial; 4 stimuli (abstract shapes) in 4 locations. In the sample phase (top row) each shape is presented individually and the monkey has to touch the shape in its ‘correct’ location to receive a food reward. After a 5 s delay, the choice phase begins in which each shape is presented in all locations. Touching the shape in the correct location results in a food pellet and the next shape is presented. Touching the shape in an incorrect location ends that attempt and initiates a 10 s time out. Another attempt at the same trial begins with the sample phase, and stimuli are presented in the same order. Monkeys are allowed up to 5 repeated attempts (i.e. 6 total attempts) before a new trial with new stimuli are presented.
(TIF)

S1 Table Response latencies in choice phase and percent task completed measures. †and †† indicate differences from vehicle condition (at *p*<0.05 and *p*<0.01 respectively). In the scopolamine dose-response study, scopolamine differed from vehicle. In the drug combination studies, vehicle+scopolamine differed significantly from vehicle+vehicle.
(DOCX)

S2 Table RM ANOVA details for choice response latency and percent task completed. n.s. = not significant (*p*>0.05).
(DOCX)

S3 Table Effects of treatments on initial correct measure. †and †† indicate vehicle+scopolamine differed significantly from vehicle+vehicle (at *p*<0.05 and *p*<0.01 respectively) after a significant RM-ANOVA. *and ** indicate EVP6124+scopolamine differed from vehicle+scopolamine (*p*<0.05 and *p*<0.05, respectively) after a significant RM-ANOVA (ANOVA details are in S4 Table). EVP-6124 exacerbated the scopolamine-induced impairment in initial attempt accuracy.
(DOCX)

S4 Table RM ANOVA details for percent correct on initial attempt. n.s. = not significant (*p*>0.05).
(DOCX)

S5 Table Plasma drug levels after i.m. administration in cynomolgus monkeys. n.d. = not determined.
(DOCX)

S1 File Supplemental data file. S1 File includes all applicable data for this manuscript.
(XLSX)
