# Supplementary figures and images for: Nicotinic alpha 7 receptor agonists EVP-6124 and BMS-933043, attenuate scopolamine-induced deficits in visuo-spatial paired associates learning
Source: PLoS One. 2017 Dec 19;12(12):e0187609. doi: 10.1371/journal.pone.0187609 (PMC5736175; doi:10.1371/journal.pone.0187609)

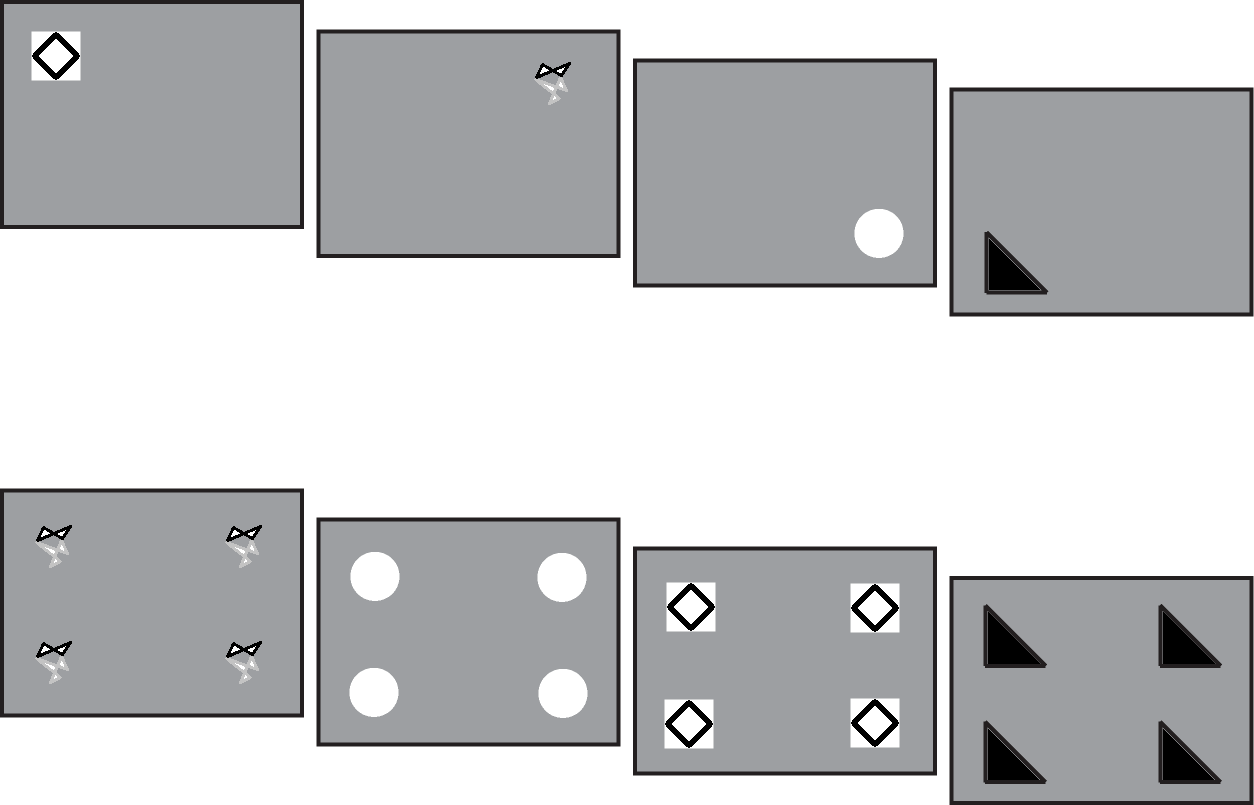

Supplement: S1 Fig — Example of the most difficult trial; 4 stimuli (abstract shapes) in 4 locations. In the sample phase (top row) each shape is presented individually and the monkey has to touch the shape in its ‘correct’ location to receive a food reward. After a 5 s delay, the choice phase begins in which each shape is presented in all locations. Touching the shape in the correct location results in a food pellet and the next shape is presented. Touching the shape in an incorrect location ends that attempt and initiates a 10 s time out. Another attempt at the same trial begins with the sample phase, and stimuli are presented in the same order. Monkeys are allowed up to 5 repeated attempts (i.e. 6 total attempts) before a new trial with new stimuli are presented. (TIF) [file pone.0187609.s001.tif]
