# Supplementary material for: Nicotinic alpha 7 receptor agonists EVP-6124 and BMS-933043, attenuate scopolamine-induced deficits in visuo-spatial paired associates learning
Source: PLoS One. 2017 Dec 19;12(12):e0187609. doi: 10.1371/journal.pone.0187609 (PMC5736175; doi:10.1371/journal.pone.0187609)
Supplement: S1 Table — †and †† indicate differences from vehicle condition (at p<0.05 and p<0.01 respectively). In the scopolamine dose-response study, scopolamine differed from vehicle. In the drug combination studies, vehicle+scopolamine differed significantly from vehicle+vehicle. (DOCX) [file pone.0187609.s002.docx]

|  | Choice Response Latency, ms (SEM) | Percent Task Completion  (SEM) |
| --- | --- | --- |
| Vehicle | 2,140 (164) | 91.5% (4%) |
| Scopolamine 0.003 mg/kg | 2,454 (540) | 93.0% (5%) |
| Scopolamine 0.0056 mg/kg | 2,244 (136) | 64.1% (10%) †† |
| Scopolamine 0.010 mg/kg | 2,151 (193) | 60.9% (8%) †† |
| Scopolamine 0.017 mg/kg | 2,671 (115) | 49.9% (5%) †† |
|  |  |  |
| Vehicle + Vehicle | 2,204 (162) | 98.8% (1%) |
| Vehicle + Scopolamine | 2,407 (152) | 77.1% (7%) † |
| Donepezil 0.03 mg/kg + Scopolamine | 2,340 (123) | 79.4% (6%) |
| Donepezil 0.10 mg/kg + Scopolamine | 2,362 (167) | 80.6% (6%) |
| Donepezil 0.30 mg/kg + Scopolamine | 2,371 (123) | 77.8% (10%) |
|  |  |  |
| Vehicle + Vehicle | 1,763 (197) | 97.4% (3%) |
| Vehicle + Scopolamine | 2,033 (150) | 82.3% (7%) † |
| BMS-933043 0.03 mg/kg + Scopolamine | 2,008 (230) | 80.9% (9%) |
| BMS-933043 0.10 mg/kg + Scopolamine | 1,959 (225) | 84.8% (6%) |
| BMS-933043 0.30 mg/kg + Scopolamine | 1,774 (149) | 86.7% (9%) |
| BMS-933043 1.00 mg/kg + Scopolamine | 2,029 (186) | 71.6% (8%) |
|  |  |  |
| Vehicle + Vehicle | 1,610 (125) | 99.8% (0%) |
| Vehicle + Scopolamine | 1,935 (150) | 83.8% (4%) † |
| EVP-6124 0.03 mg/kg + Scopolamine | 1878.3 (148) | 80.1% (5%) |
| EVP-6124 0.10 mg/kg + Scopolamine | 1,936 (146) | 76.1% (7%) |
| EVP-6124 0.30 mg/kg + Scopolamine | 2,400 (442) | 72.2% (6%) |
| EVP-6124 1.00 mg/kg + Scopolamine | 2,060 (243) | 76.5% (7%) |
|  |  |  |
| Vehicle + Vehicle | 1,528 (142) | 99.8% (0%) |
| Vehicle + Scopolamine | 2,022 (111) † | 70.4% (4%) †† |
| EVP-6124 0.003 mg/kg + Scopolamine | 2,046 (118) | 64.6% (7%) |
| EVP-61243 0.01 mg/kg + Scopolamine | 2,113 (174) | 71.1% (8%) |
|  |  |  |
| Vehicle + Vehicle | 1,812 (193) | 99.5% (0%) |
| Vehicle + Scopolamine | 2,342 (199) | 72.5% (7%) †† |
| RG3487 0.03 mg/kg + Scopolamine | 2,136 (187) | 73.4% (9%) |
| RG3487 0.10 mg/kg + Scopolamine | 2,203 (186) | 75.6% (8%) |
| RG3487 0.30 mg/kg + Scopolamine | 2,047 (162) | 74.6% (9%) |
| RG3487 1.00 mg/kg + Scopolamine | 2,200 (198) | 78.8% (9%) |
|  |  |  |
