# Supplementary material for: Nicotinic alpha 7 receptor agonists EVP-6124 and BMS-933043, attenuate scopolamine-induced deficits in visuo-spatial paired associates learning
Source: PLoS One. 2017 Dec 19;12(12):e0187609. doi: 10.1371/journal.pone.0187609 (PMC5736175; doi:10.1371/journal.pone.0187609)
Supplement: S2 Table — n.s. = not significant (p>0.05). (DOCX) [file pone.0187609.s003.docx]

| One way RM ANOVA | Choice Response Latency | Percent Task Completed |
| --- | --- | --- |
| Treatment | Main Effect | Main Effect |
| Scopolamine dose-response | F_4,28_=0.72; *p*> 0.05 | F_4,28_=11.0; *p*< 0.001 |
| Donepezil+scopolamine | F_4,20_=0.39; *p*> 0.05 | F_4,20_=3.6; *p*= 0.024 |
| BMS-933043+scopolamine | F_5,30_=1.5; *p*> 0.05 | F_5,30_=4.3; *p*= 0.004 |
| EVP-6124+scopolamine (1) | F_5,45_=1.8; *p*> 0.05 | F_5,45_=7.9; *p*<0.001 |
| EVP-6124+scopolamine (2) | F_3,18_=3.9; *p*= 0.025 | F_3,18_=12.6; *p*<0.001 |
| RG3487+scopolamine | F_5,35_=2.3; *p*> 0.05 | F_5,35_=5.0; *p*= 0.002 |
|  |  |  |
| Holm-Sidak Post-Hoc | Scopolamine differs from Vehicle | |
| Scopolamine dose-response | n.s. | 0.0056, 0.01 and 0.017 mg/kg  *p*<0.01 |
|  |  |  |
| Holm-Sidak Post-Hoc | Vehicle+Scopolamine differs from Vehicle+Vehicle | |
| Donepezil study | n.s. | *p*<0.05 |
| BMS-933043 study | n.s. | *p*<0.05 |
| EVP-6124 study (1) | n.s. | *p*<0.05 |
| EVP-6124 study (2) | *p*<0.05 | *p*<0.01 |
| RG3487 study | n.s. | *p*<0.01 |
|  |  |  |
| Holm-Sidak Post-Hoc | Treatment+Scopolamine differs from Vehicle+Scopolamine | |
| Donepezil+scopolamine | n.s. | n.s. |
| BMS-933043+scopolamine | n.s. | n.s. |
| EVP-6124+scopolamine (1) | n.s. | n.s. |
| EVP-6124+scopolamine (2) | n.s. | n.s. |
| RG3487+scopolamine | n.s. | n.s. |
