# Supplementary material for: Nicotinic alpha 7 receptor agonists EVP-6124 and BMS-933043, attenuate scopolamine-induced deficits in visuo-spatial paired associates learning
Source: PLoS One. 2017 Dec 19;12(12):e0187609. doi: 10.1371/journal.pone.0187609 (PMC5736175; doi:10.1371/journal.pone.0187609)
Supplement: S3 Table — †and †† indicate vehicle+scopolamine differed significantly from vehicle+vehicle (at p<0.05 and p<0.01 respectively) after a significant RM-ANOVA. *and ** indicate EVP6124+scopolamine differed from vehicle+scopolamine (p<0.05 and p<0.05, respectively) after a significant RM-ANOVA (ANOVA details are in S4 Table). EVP-6124 exacerbated the scopolamine-induced impairment in initial attempt accuracy. (DOCX) [file pone.0187609.s004.docx]

|  | Percent Correct on Initial Attempt (SEM) | | |
| --- | --- | --- | --- |
|  | 2 stimuli | 3 stimuli | 4 stimuli |
| Vehicle + Vehicle | 87.9% (2.7%) | 73.3% (6.2%) | 58.3% (5.7%) |
| Vehicle + Scopolamine | 79.6% (5.3%) | 49.2% (6.5%) **†** | 25.8% (4.5%) **††** |
| Donepezil 0.03 mg/kg + Scopolamine | 81.7% (7.0%) | 58.3% (7.9%) | 16.7% (6.1%) |
| Donepezil 0.10 mg/kg + Scopolamine | 78.3% (7.5%) | 63.3% (11.2%) | 30.0% (6.8%) |
| Donepezil 0.30 mg/kg + Scopolamine | 80.0% (6.8%) | 63.3% (4.2%) | 28.2% (7.2%) |
|  |  |  |  |
| Vehicle + Vehicle | 91.4% (1.9%) | 83.1% (3.7%) | 65.1% (7.8%) |
| Vehicle + Scopolamine | 89.6% (1.8%) | 67.0% (5.0%) | 37.3% (7.4%) **††** |
| BMS-933043 0.03 mg/kg + Scopolamine | 90.0% (3.1%) | 58.6% (8.0%) | 38.3% (9.8%) |
| BMS-933043 0.10 mg/kg + Scopolamine | 88.6% (3.4%) | 75.7% (5.7%) | 35.7% (8.1%) |
| BMS-933043 0.30 mg/kg + Scopolamine | 91.4% (7.0%) | 68.6% (11.2%) | 45.0% (5.6%) |
| BMS-933043 1.00 mg/kg + Scopolamine | 95.7% (2.0%) | 71.4% (5.9%) | 41.4% (8.3%) |
|  |  |  |  |
| Vehicle + Vehicle | 94.0% (1.5%) | 89.6% (3.1%) | 75.1% (4.6%) |
| Vehicle + Scopolamine | 87.3% (4.0%) | 70.0% (3.4%) **††** | 38.8% (4.0%) **††** |
| EVP-6124 0.03 mg/kg + Scopolamine | 88.8% (4.8%) | 61.3% (7.2%) | 32.5% (6.5%) |
| EVP-6124 0.10 mg/kg + Scopolamine | 91.3% (3.5%) | 58.8% (6.1%) | 25.6% (6.1%) |
| EVP-6124 0.30 mg/kg + Scopolamine | 86.7% (4.7%) | 52.2% (8.5%) ***** | 26.3% (8.0%) |
| EVP-6124 1.00 mg/kg + Scopolamine | 88.9% (4.2%) | 51.1% (9.3%) ****** | 28.9% (6.0%) |
|  |  |  |  |
| Vehicle + Vehicle | 94.6% (2.4%) | 87.1% (5.3%) | 76.8% (6.%) |
| Vehicle + Scopolamine | 91.9% (3.6%) | 66.2% (7.6%) **†** | 34.6% (4.2%) **††** |
| EVP-6124 0.003 mg/kg + Scopolamine | 75.7% (8.1%) | 60.% (12.7%) | 20.0% (7.7%) |
| EVP-61243 0.01 mg/kg + Scopolamine | 82.9% (6.1%) | 65.7% (13.1%) | 28.3% (9.8%) |
|  |  |  |  |
| Vehicle + Vehicle | 92.1% (2.5%) | 80.8% (5.5%) | 66.6% (8.1%) |
| Vehicle + Scopolamine | 84.3% (2.8%) | 46.2% (8.0%) **††** | 19.5% (4.9%) **††** |
| RG3487 0.03 mg/kg + Scopolamine | 83.8% (4.6%) | 43.8% (10.5%) | 25.0% (7.8%) |
| RG3487 0.10 mg/kg + Scopolamine | 90.0% (3.3%) | 47.5% (8.0%) | 13.8% (6.5%) |
| RG3487 0.30 mg/kg + Scopolamine | 83.8% (7.1%) | 42.5% (9.0%) | 20.4% (7.5%) |
| RG3487 1.00 mg/kg + Scopolamine | 77.5% (6.2%) | 48.8% (8.3%) | 22.2% (5.6%) |
|  |  |  |  |
