# Supplementary material for: Nicotinic alpha 7 receptor agonists EVP-6124 and BMS-933043, attenuate scopolamine-induced deficits in visuo-spatial paired associates learning
Source: PLoS One. 2017 Dec 19;12(12):e0187609. doi: 10.1371/journal.pone.0187609 (PMC5736175; doi:10.1371/journal.pone.0187609)
Supplement: S4 Table — n.s. = not significant (p>0.05). (DOCX) [file pone.0187609.s005.docx]

| 2-Factor RM ANOVA | Factor 1 | Factor 2 |  |
| --- | --- | --- | --- |
| Treatment | Difficulty | Treatment | Interaction |
| Donepezil+scopolamine | F_3,15_=81.2; *p*< 0.001 | F_4,20_=3.5; *p*= 0.025 | F_12,60_=2.6; *p*= 0.008 |
| BMS-933043+scopolamine | F_3,18_=158.4; *p*< 0.001 | F_5,30_=2.1; *p*= 0.093 | F_15,90_=1.5; *p*= 0.12 |
| EVP-6124+scopolamine (1) | F_3,27_=199.4; *p*< 0.001 | F_5,45_=14.0; *p*< 0.001 | F_15,135_=5.9; *p*< 0.001 |
| EVP-6124+scopolamine (2) | F_3,18_=45.7; *p*< 0.001 | F_3,18_=18.7; *p*< 0.001 | F_9,54_=3.7; *p*= 0.001 |
| RG3487+scopolamine | F_3,21_=113.0; *p*< 0.001 | F_5,35_=11.0; *p*< 0.01 | F_15,105_=4.1; *p*< 0.01 |
|  |  |  |  |
| Holm-Sidak Post-Hoc | Negative Control (Vehicle+Scopolamine) differs from Vehicle+Vehicle | | |
|  | 2-stimuli | 3-stimuli | 4 stimuli |
| Donepezil study | n.s. | *p*<0.05 | *p*<0.01 |
| BMS-933043 study | n.s. | n.s. | *p*<0.01 |
| EVP-6124 study (1) | n.s. | *p*<0.01 | *p*<0.01 |
| EVP-6124 study (2) | n.s. | *p*<0.05 | *p*<0.01 |
| RG3487 study | n.s. | *p*<0.01 | *p*<0.01 |
|  |  |  |  |
| Holm-Sidak Post-Hoc | Treatment+Scopolamine differs from Vehicle+Scopolamine | | |
|  | 2-stimuli | 3-stimuli | 4 stimuli |
| Donepezil+scopolamine | n.s. | n.s. | n.s. |
| BMS-933043+scopolamine | n.s. | n.s. | n.s. |
| EVP-6124+scopolamine (1) | n.s. | 1. mg/kg *p*<0.01   Further impaired  0.3 mg/kg *p*<0.05  Further impaired | n.s. |
| EVP-6124+scopolamine (2) | n.s. | n.s. | n.s. |
| RG3487+scopolamine | n.s. | n.s. | n.s. |
