# Supplementary material for: Nicotinic alpha 7 receptor agonists EVP-6124 and BMS-933043, attenuate scopolamine-induced deficits in visuo-spatial paired associates learning
Source: PLoS One. 2017 Dec 19;12(12):e0187609. doi: 10.1371/journal.pone.0187609 (PMC5736175; doi:10.1371/journal.pone.0187609)
Supplement: S5 Table — n.d. = not determined. (DOCX) [file pone.0187609.s006.docx]

|  | nM total compound in plasma post session,  (mean 69.8 min post injection; SD=9.8 min) | nM total compound in plasma from  satellite animals, 60 min |
| --- | --- | --- |
| Scopolamine 0.003 mg/kg | n.d. | 2.8 |
| Scopolamine 0.0056 mg/kg | n.d. | 2.9 |
| Scopolamine 0.010 mg/kg | n.d. | 12.0 |
| Scopolamine 0.017 mg/kg | n.d. | 14.6 |
|  |  |  |
| Donepezil 0.03 mg/kg | n.d. | 7.4 |
| Donepezil 0.10 mg/kg | n.d. | 19.2 |
| Donepezil 0.30 mg/kg | n.d. | 90.3 |
|  |  |  |
| BMS-933043 0.03 mg/kg | 13.3 (1.1) | 18.4 |
| BMS-933043 0.10 mg/kg | 47.8 (7.9) | 65.9 |
| BMS-933043 0.30 mg/kg | 139.5 (13.2) | 186.0 |
| BMS-933043 1.00 mg/kg | 492.8 (97.7) | 919.5 |
|  |  |  |
| EVP-6124 0.003 mg/kg | 1.0 (0.51) | n.d. |
| EVP-61243 0.01 mg/kg | 1.6 (0.33) | n.d. |
| EVP-6124 0.03 mg/kg | 5.1 (1.4) | 3.7 |
| EVP-6124 0.10 mg/kg | 14.1 (2.8) | 9.9 |
| EVP-6124 0.30 mg/kg | 53.1 (14.4) | 44.2 |
| EVP-6124 1.00 mg/kg | 170.2 (30.4) | 105.5 |
|  |  |  |
| RG3487 0.03 mg/kg | n.d. | n.d. |
| RG3487 0.10 mg/kg | n.d. | 58.7 |
| RG3487 0.30 mg/kg | n.d. | 123.0 |
| RG3487 1.00 mg/kg | n.d. | 450.0 |
